# Mammalian FMRP S499 Is Phosphorylated by CK2 and Promotes Secondary Phosphorylation of FMRP

**DOI:** 10.1523/ENEURO.0092-16.2016

**Published:** 2016-11-21

**Authors:** Christopher M. Bartley, Rachel A. O’Keefe, Anna Blice-Baum, Mihaela-Rita Mihailescu, Xuan Gong, Laura Miyares, Esra Karaca, Angélique Bordey

**Affiliations:** 1 Departments of Neurosurgery, and Cellular and Molecular Physiology, Yale University School of Medicine, New Haven, CT 06510; 2 Department of Neurobiology, Yale University School of Medicine, New Haven, CT 06510; 3 Department of Chemistry and Biochemistry, Duquesne University, Pittsburgh, PA 15282; 4 Department of Neurosurgery, Xiangya Hospital, Central South University, Changsha 410008, China

**Keywords:** casein kinase, FMRP, fragile X, mTOR, phosphorylation, translation

## Abstract

The fragile X mental retardation protein (FMRP) is an mRNA-binding regulator of protein translation that associates with 4-6% of brain transcripts and is central to neurodevelopment. Autism risk genes’ transcripts are overrepresented among FMRP-binding mRNAs, and FMRP loss-of-function mutations are responsible for fragile X syndrome, the most common cause of monogenetic autism. It is thought that FMRP-dependent translational repression is governed by the phosphorylation of serine residue 499 (S499). However, recent evidence suggests that S499 phosphorylation is not modulated by metabotropic glutamate receptor class I (mGluR-I) or protein phosphatase 2A (PP2A), two molecules shown to regulate FMRP translational repression. Moreover, the mammalian FMRP S499 kinase remains unknown. We found that casein kinase II (CK2) phosphorylates murine FMRP S499. Further, we show that phosphorylation of FMRP S499 permits phosphorylation of additional, nearby residues. Evidence suggests that these nearby residues are modulated by mGluR-I and PP2A pathways. These data support an alternative phosphodynamic model of FMRP that is harmonious with prior studies and serves as a framework for further investigation.

## Visual Abstract

**Figure:**
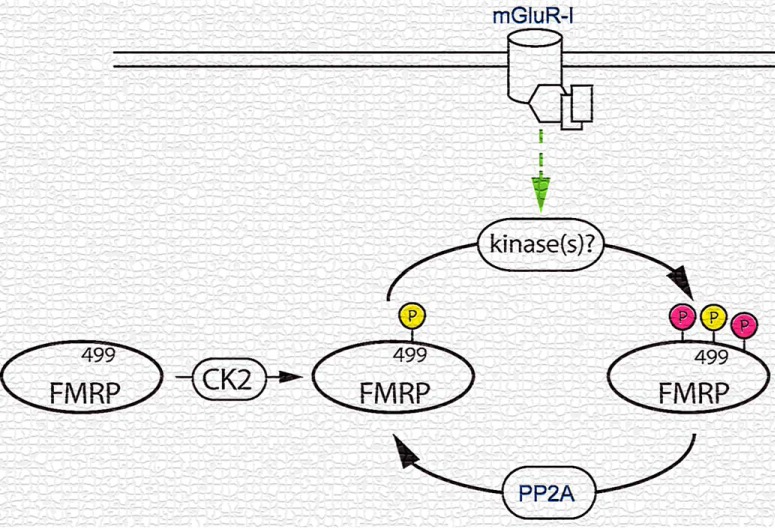

## Significance Statement

FMRP’s role in translation and plasticity is dependent on the phosphorylation status of serine 499 (S499); however, the kinase phosphorylating FMRP remains unknown in mammals. Here, we identified that a constitutively active kinase, casein kinase II (CK2), phosphorylates mammalian FMRP S499. In addition, S499 phosphorylation allows phosphorylation of additional, nearby residues that are likely modulated downstream of metabotropic glutamate signaling in an activity-dependent manner. This finding provides an explanation for how FMRP S499 contributes to activity-dependent FMRP function without itself being modulated by activity.

## Introduction

FMRP is an mRNA-binding protein that regulates the translation of 4–6% of brain mRNAs, many of which are involved in neuroplasticity (Ascano et al., 2012; Brown et al., 2001; Darnell et al., 2011). CGG expansion of exon 1 of the *FMR1* gene (the X-linked gene encoding FMRP) results in fragile X syndrome (FXS), the leading cause of inherited intellectual disability and monogenetic autism (Eberhart and Warren, 1996). Moreover, mRNA transcripts implicated in autism and other neuropsychiatric disorders are overrepresented in the population of FMRP-interacting mRNAs, further highlighting FMRP’s role in neurocognitive function (Iossifov et al., 2012; Fromer et al., 2014). Under normal conditions, FMRP’s contribution to neuroplasticity is in part dictated by phosphorylation of serine 499 (S499 in mouse, S500 in humans), resulting in FMRP associating with stalled polyribosomes and translational repression (Ceman et al., 2003; Mazroui et al., 2003). That the phosphorylation of this site is required for rescue of fly models of FXS demonstrates its requisite role in FMRP biology (Coffee et al., 2012). In spite of the importance of this single residue, remarkably little is known about the phosphoregulation of this site in mammals, including its phosphorylating kinase (Bartley et al., 2014).

Others have shown that downstream of mGluR class I signaling (mGluR-I), the phosphorylation status of this site influences translation of FMRP-associated transcripts as well as FMRP ubiquitination and degradation. These observations, among others, underlie the dominant model that mGluR-I signaling mediates dynamic changes in the phosphorylation of FMRP S499 and subsequent translational derepression. At odds with this model are studies showing that, with rare exceptions (Muddashetty et al., 2011), the function of phosphomimetic FMRP S499 (aspartic acid mutant, FMRP D499) is statistically indistinguishable from that of wild-type FMRP S499 (Ceman et al., 2003; Konishi et al., 2004; Coffee et al., 2012; Lee et al., 2011; Nalavadi et al., 2012; Niere et al., 2012). Using site-nonspecific radioactive phosphate, previous studies have shown that FMRP’s overall phosphorylation status is modulated by the mGluR-I agonist (S)-3,5-dihydroxyphenylglycine (DHPG; Narayanan et al., 2007, 2008); however, a recent study used a site-specific antibody to show that the proportion of FMRP phosphorylated at S499 was not affected by DHPG (Bartley et al., 2014). A possible alternative model of FMRP function is that constitutive phosphorylation of S499 by an unknown kinase is necessary for the activity-dependent phosphorylation of other residues. Such a mechanism would be consistent with the model holding that mGluR-I–dependent phosphorylation regulates FMRP function. This model served as the hypothesis motivating this study.

In *Drosophila*, casein kinase II (CK2) phosphorylates FMRP on a putatively homologous residue (S406; Siomi et al., 2002). In contrast, Narayanan et al. (2008) concluded that CK2 does not phosphorylate S499 in cultured hippocampal mouse neurons. Notably, that study used a single, low-affinity CK2 antagonist. As such, it remains unknown whether CK2 also phosphorylates FMRP S499 in mammalian cells. Here, we provide evidence that CK2 phosphorylates S499 in murine and human cells, thereby reconciling the FMRP insect literature with mammalian data. However, this raises a functional issue, as CK2 is considered to be constitutively active (Ruzzene et al., 2010), and its kinase activity is not regulated by neuronal activity. FMRP S499 could be regulated by dephosphorylation alone; however, it has been shown that S499 phosphorylation levels are not affected by PP2A, the putative FMRP phosphatase (Bartley et al., 2014). A recent exhaustive study has shown that although CK2 is constitutively active, it can promote secondary hierarchical phosphorylation by other kinases, many of which are regulated in a signal-dependent manner (St-Denis et al., 2015). This supported our hypothesis that phosphorylation of FMRP S499 is permissive for secondary phosphorylation of FMRP. Using S499 phosphomutants and both an immunoaffinity and mass spectrometry approach, we determined that S499 phosphorylation is indeed permissive for the phosphorylation of additional FMRP serine or threonine residues. Finally, we report evidence that both activation of mGluR-I and inhibition of PP2A increased the degree of phosphorylation of additional FMRP residues in an S499-dependent manner. These data provide a possible explanation for how FMRP S499 contributes to activity-dependent FMRP function without itself being modulated by activity. Collectively, these findings provide a testable framework for further investigation of how neuronal activity regulates FMRP phosphorylation to modulate neuroplasticity.

## Materials and Methods

### Kinase prediction and kinase assays

Kinase predictions were performed using phosphonet.ca or the iGPS kinase prediction platform (Xue et al., 2008; Song et al., 2012). Kinase assays were performed by Kinexus Bioinformatics (http://www.kinexus.ca, Vancouver) using human wild-type recombinant FMRP S500 (rFMRP, 11.7-mm stock). Quality control testing was performed on each of the kinases to ensure compliance to acceptable standards. Kinases assays were performed on two separate occasions. After termination of the reaction with 1× Laemmli buffer, the samples were separated by SDS-PAGE and probed with phospho FMRP (pFMRP) antibody. Protein kinase assays were performed at ambient temperature for 30 min in a final volume of 25 µl according to the following assay reaction recipe: Component 1, 5 µl of diluted active protein kinase target (∼10–50 nm final protein concentration in the assay); component 2, 5 µl of test substrate FMRP S500 (11.7 µm); component 3, 10 µl of kinase assay buffer (25 mm 3-(N-morpholino)propanesulfonic acid, pH 7.2; 12.5 mm β-glycerophosphate, 25 mm MgCl_2_, 5 mm EGTA, 2 mm EDTA, and 0.25 mm dithiothreitol added to the kinase assay buffer just before use); and component 4, 5 µl of cold ATP (250-µm stock solution). The assay was initiated by the addition of ATP, and the reaction mixture was incubated at ambient temperature for 30 min. A blank control was set up for each protein kinase, which included all assay components except rFMRP (replaced with equal volume of assay dilution buffer). The corrected activity for each kinase target was determined by removing the blank control value.

### In-house CK2 kinase assay with rFMRP

rFMRP was diluted to 11.7 µm in CK2 kinase buffer (NEBuffer for Protein Kinases, #B6022; New England Biolabs, Ipswich, MA) and incubated with 4 mm ATP with or without active CK2 (New England Biolabs, #P6010). Reactions (25-µl) were performed according to the manufacturer’s protocol (New England Biolabs). Human recombinant FMRP S500 was generated as previously described (Evans and Mihailescu, 2010).

### N2a and HEK293 cell culture

Unless otherwise mentioned, murine Neuro-2a (N2a; ATCC, Manassas, VA, cat. #CCL-131, RRID:CVCL_0470**)** and HEK293 cells (gift from Sklar laboratory, Yale University) were cultured in complete medium [Dulbecco’s modified Eagle medium (DMEM); #11965-092; Invitrogen, San Diego, CA, and 5% fetal bovine serum (FBS; 16140-071; Invitrogen] in a 37°C incubator at 5% CO_2_. When cells reached approximately 70% confluence in six-well plates, pharmacologic treatment was performed. Cell lysis was performed on ice. Cells were rinsed twice with ice-cold 1× PBS and lysed in N2a lysis buffer [radioimmunoprecipitation assay buffer (RIPA), 1× Halt protease/phosphatase inhibitor cocktail, Thermo Fisher Scientific, Waltham, MA, #78440; 8 U/10 ml DNase I, Roche, Basel, Switzerland, #04716728001; 100 nm okadaic acid, Tocris Bioscience, Bristol, UK, #1136]. Cells were then scraped from the wells, and lysates were centrifuged at 16,000 relative centrifugal force (rcf) for 10 min at 4°C. The supernatant was added to 6× Laemmli sample buffer to a final concentration of 1× sample buffer and boiled for 5 min at 99°C.

### Animals and primary mouse cortical neuron cultures

All animal procedures were performed in accordance with the Yale University Institutional Animal Care and Use Committee. The following procedure was performed on CD-1 mice (Charles River Laboratories, Wilmington, MA) of either sex. The cortices of embryonic day-16 mouse pups were dissected out, incubated in papain (Worthington, Freehold, NJ) for 15 min at 37°C, and dissociated by pipetting in plating medium [minimum essential medium (MEM) supplemented with 5% fetal calf serum, 0.6% glucose, and 2 mm GlutaMAX]. After dissociation, cells were resuspended in MEM supplemented with 0.6% glucose and 5% fetal bovine serum and plated on poly-d-lysine–coated six-well plates. The medium was changed to neuronal maintenance media (neurobasal medium with 1× B27 and 1× GlutaMAX-1) 1–2 h after plating. Half of the medium was then changed every 3 days. Neurons were plated at 10^6^ cells per well, and pharmacologic treatments were performed 6 d after plating.

### Antibodies

Antibodies and usage parameters are listed in Table 1.

**Table 1.** List of antibodies.

Antibody	Manufacturer (species, cat. no.)	Concentrations (primary; secondary)	Blocking solution
AKT	Cell Signaling Technology; 4685 RRID:AB_2225340	1:5000; 1:5000	5% Milk/TBST
pERK T202/Y204	Cell Signaling Technology; 4370 RRID:AB_2315112	1:10,000; 1:5000	5% BSA/TBST
ERK	Santa Cruz Biotechnology; sc-94 RRID:AB_2140110	1:20,000; 1:10,000	5% Milk/TBST
pFMRP S499	PhosphoSolutions; p1125-499 RRID:AB_2492094	1:1000; 1:2000	Block milk 5%, probe in BSA (pFMRP must be probed for before FMRP)
FMRP	Abcam; ab17722 RRID:AB_2278530	1:5000; 1:5000	5% Milk/TBST
pS6 S240/244	Cell Signaling Technology; 5364 RRID:AB_10694233	1:10,000; 20,000	5% BSA/TBST (total S6 must be probed for first, as pS6 is not efficiently stripped)
Total S6	Cell Signaling Technology; 2217 RRID:AB_331355	1:5000; 1:10,000	5% Milk/TBST
Phosphoserine antibody	Millipore; AB1603 RRID:AB_390205	1:2000; 1:2000	See Protocols.io
Phosphothreonine HRP conjugate antibody	Cell Signaling Technology; 6949S RRID:AB_10828224	1:1000; no secondary	See Protocols.io
Phosphotyrosine antibody	Cell Signaling Technology (Rb) #8954	1:1000; 1:2000	See Protocols.io
Goat anti-mouse IgG–HRP secondary	Santa Cruz Biotechnology; sc-2005 RRID:AB_631736	1:10,000	5% Milk/TBST
Anti-rabbit IgG secondary	Cell Signaling Technology; 7074 RRID:AB_2099233	1:5000	5% Milk/TBST

### Plasmid and transfection

Transfections were formed using PolyJet transfection reagent (SignaGen) according to the manufacturer’s protocol using 2× recommended DNA concentrations. Vectors included pEGFP-C1 (Clontech, Cambridge, UK) and N-terminal GST-tagged murine FMRP (GST-FMRP) from Dr. Xinyu Zhao, University of Wisconsin, Madison, WI, with additional modifications as follows: S499A and S499D.

### Immunoblotting

All Western blots were performed using Bio-Rad 10% Tris-glycine gels and protein transferred to PVDF membranes according to a standard wet transfer protocol. In general, the optimal linear range for each antibody was determined before experimental immunoblot assays. In some cases, limited linear ranges were run on the same gel (that is, 80% and 120% of a control sample were loaded in end lanes) to ensure detectability of minor changes in protein signals. Densitometry was performed using ImageJ without background correction or rolling ball adjustments. All phosphoprotein signals were normalized to total protein signals from the same blot. For phosphoproteins, adequate removal of phosphoantibody was verified by probing with secondary antibody alone after stripping the membrane. Raw ratios of phosphoprotein normalized to total protein or total protein normalized to loading control (generally ERK 1/2 unless otherwise stated) were calculated in Microsoft Excel, and statistical tests were performed in GraphPad Prism 6 (RRID:SCR_002798**)**.

### CK2 washout assay

For the washout assay, lysates from N2a cells were first collected at time 0 to obtain baseline FMRP S499 levels. N2a cells were then incubated with either CX-4945 (5 µm) or an equal volume of dimethylsulfoxide. After 24 h, a set of N2a cells’ lysates was collected to establish baseline FMRP S499 in each condition after 24 h of treatment. In the remaining wells, the medium was replaced with fresh medium, and N2a cells’ lysates were collected at various intervals (5, 15, 30, 60, or 180 min).

### Immunoprecipitations of recombinant GST-FMRP

#### Immunoprecipitation for immunoblotting

On the day of immunoprecipitation, N2a cells in six-well plates were placed on ice and rinsed with ice-cold 1× PBS twice. PBS was aspirated, and 300 µl of N2a lysis buffer was added to each well. After 2 min, well bottoms were scraped with the back of a pipet tip to manually aid in cell lysis. Two wells per condition were combined into a single 1-ml Eppendorf tube for a total of 600 µl cell lysate. Lysates were centrifuged at 13,200 rcf for 10 min at 4°C. Supernatant (75 µl) was transferred to a new tube to which an equal volume of sample buffer was added, boiled for 5 min at 99°C, and stored as input. For IP reactions, the remaining ∼500 µl of supernatant was transferred to a new tube to which 500 µl IP buffer was added (NaCl 1 m, RIPA, 2× Halt protease phosphatase inhibitor, DNase I 8 U/10 mL, okadaic acid 100 nm, and EDTA 50 mm). To each IP reaction, 40 µl anti-GST [s-tag-05] antibody was added. To each IP reaction, 50 µl protein A agarose beads were added. IP reactions were rotated overnight in a cold room at 4°C. The next day, the IP reaction was centrifuged at 10,000 rcf for 1 min. IP reaction supernatant (100 µl) was saved as flow through. The beads were rinsed with 1 m NaCl wash buffer for 15 min at 4°C. Rinses were repeated three times, with a final rinse in 120 mm NaCl wash buffer. Immunoprecipitated proteins were eluted by adding 60 µL of 2× SDS sample buffer. Samples were then boiled at 99°C for 5 min and stored at –20°C.

#### Immunoprecipitation for phosphoproteomics

N2a cells were grown in 10% fetal bovine serum/DMEM high glucose and transfected at 40% confluence. Each T75 flask was transfected with 18 µg GST-FMRP plasmid overnight using the PolyJet transfection reagent. Medium was replaced with fresh 10% FBS/DMEM the next morning, and cells were grown for an additional 48 h. Before cell lysis, 400 µl (40 µg) GST S-tag-05 (Santa Cruz Biotechnology, Santa Cruz, CA) was conjugated to 100 µl protein A beads (Thermo Fisher Scientific) for 2.5 h at 4°C. Cells were lysed at ∼90% confluence in 3 ml lysis buffer/flask (RIPA, 1× Halt phosphatase/protease inhibitor cocktail, 50 mm EDTA, 100 nm Okadaic Acid, 8U/10 ml DNase I). Samples were vortexed and centrifuged at 16,000 rcf for 10 min at 4°C. Lysates were rotated with antibody-conjugated beads, and 5 m NaCl was added to samples to bring the final NaCl concentration to 1 m. Samples were incubated on a rotator overnight at 4°C. Samples were washed twice with 1 m NaCl + 1× Halt followed by three washes with RIPA + 1× Halt. Sample buffer (60 µl, 1×) was added to each sample and boiled at 95°C for 5 min to elute proteins.

### Mass spectrometry

The phosphopeptide enrichment step and the subsequent tandem mass spectrometry were performed at the MS & Proteomics Resource at Yale University. Briefly, Coomassie-stained gel bands corresponding to the FMRP protein were in-gel digested with trypsin (MS grade, Promega, Madison, WI; incubation at 37°C overnight); peptides were extracted using an 80% acetonitrile solution containing 0.1% formic acid. Macro-spin desalt of the digests was performed using C18 spin columns (Nest Group, Southborough, MA), followed by dissolution in 3 µl of formic acid and 40–100 µl (depending on amount of sample) of a solution containing 0.5% trifluoroacetic acid and 50% acetonitrile. To isolate the phosphopeptides, the desalted digested peptide suspensions were loaded onto TiO_2_ columns Spin Cartridge (Glygen Corp., Columbia, MD), washed twice with buffer A (0.5% trifluoroacetic acid/50% acetonitrile), and eluted using a diluted (1:33) ammonia solution. The eluates were dried in a speed-vac, washed twice with 30 µl of water, and finally resuspended in 50 mm triethylammonium bicarbonate. Liquid chromatography/tandem mass spectrometry (LC-MS/MS) was performed on an LTQ Orbitrap Elite (Thermo Fisher Scientific) equipped with a Waters Symmetry C18 (180 µm × 20 mm) trap column and a 1.7 µm, 75 µm, × 250 mm nanoAcquity UPLC column (35°C). Trapping was done using 99% buffer A (100% water, 0.1% formic acid), and peptide separation was undertaken using a linear gradient of solvents A (0.1% formic acid in water) and B (0.075% formic acid in acetonitrile) over 90 min, at a flow rate of 300 nl/min. MS spectra were acquired in the Orbitrap using one microscan and a maximum injection time of 900 ms followed by three data-dependent MS/MS acquisitions in the ion trap (with precursor ions threshold of >3000). The total cycle time for both MS and MS/MS acquisition was 2.4 s. Peaks targeted for MS/MS fragmentation by collision induced dissociation (CID) were first isolated with a 2-Da window followed by normalized collision energy of 35%. Dynamic exclusion was activated where former target ions were excluded for 30 s. The raw data files were processed with Mascot Distiller (Matrix Science) and searched with in-house Mascot Search Engine (version 2.2.0) against the entire Mascot database and again using a custom FMRP FASTA protein database. The data was searched using the following search parameters: enzyme, trypsin (note that Lys C cuts after Lys and trypsin after both Lys and Arg); variable modifications, carbamidomethyl (Cys), oxidation (Met), and phosphorylation on Ser, Thr, and Tyr; mass values, monoisotopic; protein mass: unrestricted; peptide mass tolerance, ±20 ppm; fragment mass tolerance, ±0.6 Da; charge, +6; maximum missed cleavages, three; decoy, yes; instrument type: ESI-TRAP. Sites of phosphorylation were validated manually.

### Pharmacologic agents

Pharmacologic agents including their targets and suppliers are listed in Table 2.

**Table 2.** Agents used in N2a-based pharmacological screens.

Agent	Primary Target	Other targets	Suppliers
Rapamycin	mTORC1	mTORC2	A.G. Scientific, R-1018
Bisindolylmaleimide V (B5)	S6K1	Unknown	Enzo Life, ALX-270-053 Sciences
CX-4945	CK2	DYRK2	Selleckchem, S2248
TBB	CK2	DYRK2, PIM	Tocris, 2275
DRB	CDK7/CK2	CDK8, CDK9	EMD Millipore, 287891
PHA-767491	Cdc7	CDK9, MK2	Tocris, 3140
D4476	CK1	ALK5, PKD1, p38a	Tocris, 2902
IC261	CK1	PKA. Fyn	EMD Millipore, 400090
Staurosporine	Pan Kinase	PP1	Tocris, 1285

### Statistical analysis

Statistical analysis was performed on raw densitometric ratios using GraphPad Prism 6. For data presentation, values were normalized to control data such that control groups were always equal to 1. For *in vitro* experiments, data are represented as the percentage change from the control lane on the same membrane; as such, control lanes are without error bars. Statistical significance was determined using the Mann–Whitney U test, nonparametric one-way ANOVA, or two-way ANOVA using appropriate post hoc tests where indicated. *p* < 0.05 or 0.05 was considered significant. Data are shown as mean ± SEM unless otherwise specified.

## Results

### *In vitro* identification of FMRP S499 kinases

**Figure 1.**
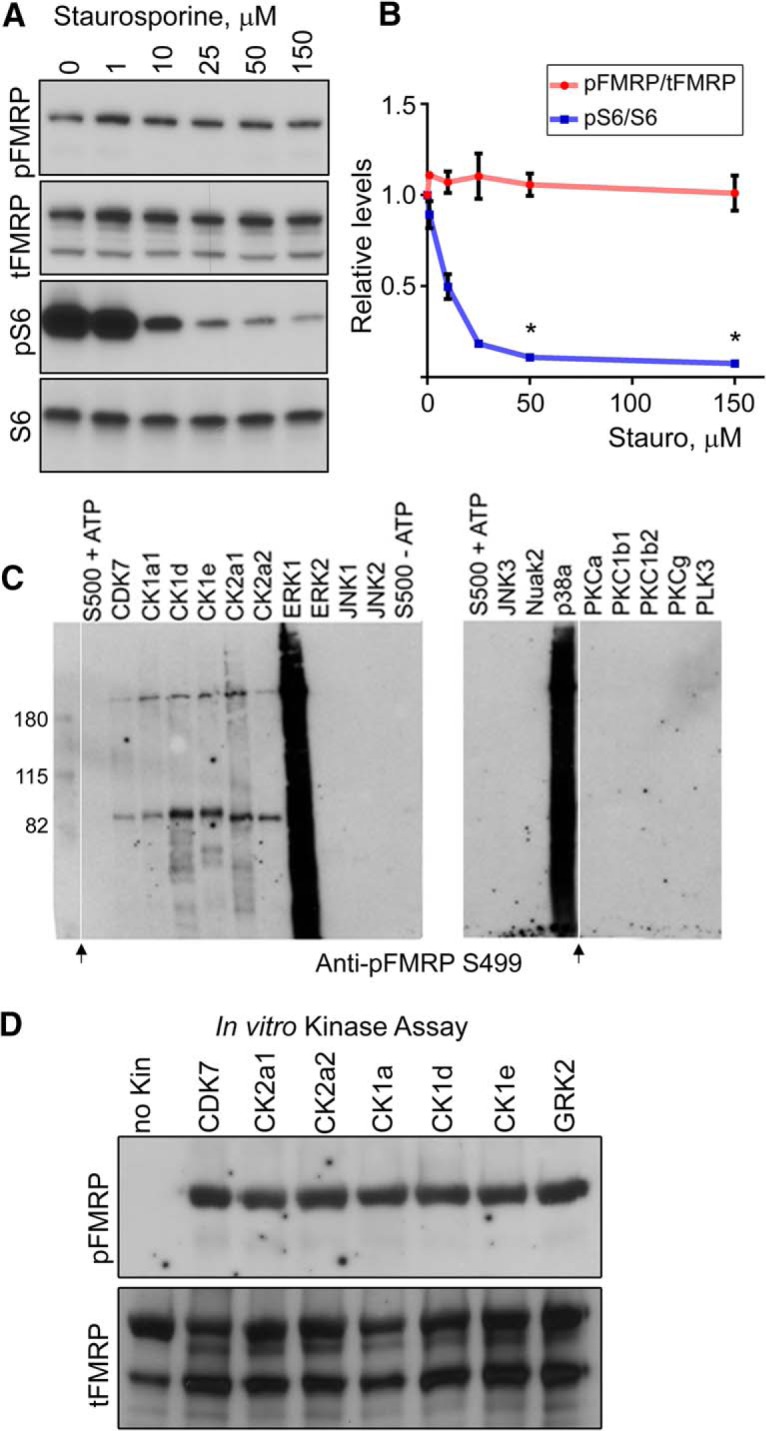
FMRP S499 is phosphorylated by multiple kinases *in vitro*. ***A***, FMRP S499 phosphorylation is not altered by 3-h staurosporine treatment. N2a cells were treated for 3 h with increasing doses of staurosporine. Staurosporine decreased rpS6 S240/244 phosphorylation without affecting FMRP S499 phosphorylation. ***B***, Quantification of rpS6 S240/244 (pS6/S6, blue) and FMRP S499 phosphorylation after staurosporine treatment. There was no significant effect of staurosporine treatment on FMRP phosphorylation (Kruskal–Wallis one-way ANOVA [*H*(5) = 2.32, *p* = 0.8034]), but there was a significant effect on rpS6 S240/244 phosphorylation (Kruskal–Wallis one-way ANOVA [*H*(5) = 16.04, *p* = 0.0067]. Post hoc Dunn’s test for multiple comparisons showed *p* < 0.05 for 50- and 150-µm treatment groups compared with 0 µm. *n* = 3, error bars = SEM. ***C***, Initial Kinexus kinase assay. rFRMP S500 was incubated with recombinant kinase listed above the SDS-PAGE on 10% Tris-glycine gels. Samples were run on two independent blots. The leftmost lane is the ladder. Arrows indicate where intervening lanes have been removed for clarity. ***D***, Repeat kinase assay including GRK2. The two panels are from the same membrane. After transfer, the membrane was probed with anti-pFMRP S499, stripped, and reprobed with tFMRP.

To narrow down the number of potential FMRP S499 kinases, we first chose to test the broad kinase inhibitor staurosporine (*Streptomyces* sp.), which inhibits close to half of the kinome (Karaman et al., 2008). Surprisingly, a 3-h-long staurosporine treatment of Neuro2a (N2a) cells (a cell line validated for the biochemical investigation of FMRP) did not significantly reduce FMRP S499 phosphorylation at any of the tested concentrations (Kruskal–Wallis one-way ANOVA, *H*(5) = 2.32, *p* = 0.8034, *n* = 3; Fig. 1*A*, *B*). Staurosporine activity was verified by a significant reduction in phosphorylation of ribosomal protein S6 (rpS6) at S240/244, a protein that is phosphorylated by the staurosporine-sensitive p70 S6 kinase (Kruskal–Wallis one-way ANOVA, H(5) = 16.04, *p* = 0.0067, *p* < 0.05 for 50 µM, and 150 µM with post hoc Dunn’s test for multiple comparisons, *n* = 3; Fig. 1*A*, *B*).

Because the staurosporine-insensitive kinases cannot be easily categorized into pharmacological subgroups, we switched to a candidate kinase approach. Candidate kinases capable of phosphorylating FMRP S499 were initially identified using two *in silico* kinase prediction platforms, phosphonet.ca and iGPS. From this list, we chose to test the top-ranked kinases available in the Kinexus recombinant kinase library (Table 3). Added to this candidate group were kinases known to be involved in synaptic plasticity (e.g., ERKs and JNKs) as well as CK2 isoforms α1 and α2, given that CK2 phosphorylates a putative homologous serine on *Drosophila* FMRP (S406; Siomi et al., 2002). Notably, JNKs have recently been shown to regulate mGluR-I–dependent protein translation, and although the authors speculated that JNK did not phosphorylate FMRP directly, this was not tested (Schmidt et al., 2013). We screened this combined set of 18 recombinant kinases against recombinant human FMRP (rFMRP, with homologous residue S500) using a validated site-specific antibody from PhosphoSolutions, Aurora, CO (Ab-pFMRP^S499^; Bartley et al., 2014; Reynolds et al., 2015). For site-specific kinase assays, rFMRP was incubated with each recombinant kinase, and ATP and then resolved by SDS-PAGE (see Methods, Kinexus kinase assay). Immunoblotting with Ab-pFMRP^S499^ showed that six of 18 kinases tested were capable of phosphorylating rFMRP S500 *in vitro*: CDK7, CK1a, CK1d, CK1e, CK2a1, and CK2a2 (Fig. 1*C*). ERK1 and p38a activity toward rFMRP could not be determined by SDS-PAGE because of artifacts. Notably, ERK1 is inhibited by staurosporine at the concentrations tested earlier. To further verify these results, the kinase assays were repeated for kinases giving a positive signal with the addition of the acidic kinase GRK2, a top-ranked S499 candidate that later became available in the Kinexus kinase library. All six kinases as well as GRK2 produced a positive Ab-pFMRP^S499^ signal (Fig. 1*D*). Consistent with our finding that FMRP S499 phosphorylation is staurosporine insensitive, the seven kinases that phosphorylated rFMRP are relatively resistant to staurosporine according to the kinase inhibitor database (in red, Table 3, ReactionBiology.com database). Collectively, these data indicate that rFMRP can be phosphorylated *in vitro* by multiple kinases at a site that is homologous to murine FMRP S499.

**Table 3.** Kinases tested in the kinase assay.

Kinase	Rank (phosphonet.ca)	Inhibition by staurosporine (%)
CK1a*	1	20.29
CK1d*	10	8.77
CK1e*	11	21.79
CK2a1*	NA	–0.64
CK2a2*	NA	33.38
CDK7*	2	56.22
PKCg	6	103.22
PKCb	9	95.87
PKCa	15	98.77
PLK3	14	62.21
GRK2*	13	44.18
Nuak2	4	79.77
Erk1	NA	2.79
Erk2	NA	2.55
Jnk1	NA	–2.48
Jnk2	NA	0.00
Jnk3	NA	–23.23

The degree to which each kinase’s activity is inhibited by staurosporine is shown in the right column (according to ReactionBiology.com). *Kinase activity toward rFMRP S500 based on SDS-PAGE. NA, not applicable.

### Evidence that CK2 phosphorylates FMRP S499 in N2a cells

**Figure 2.**
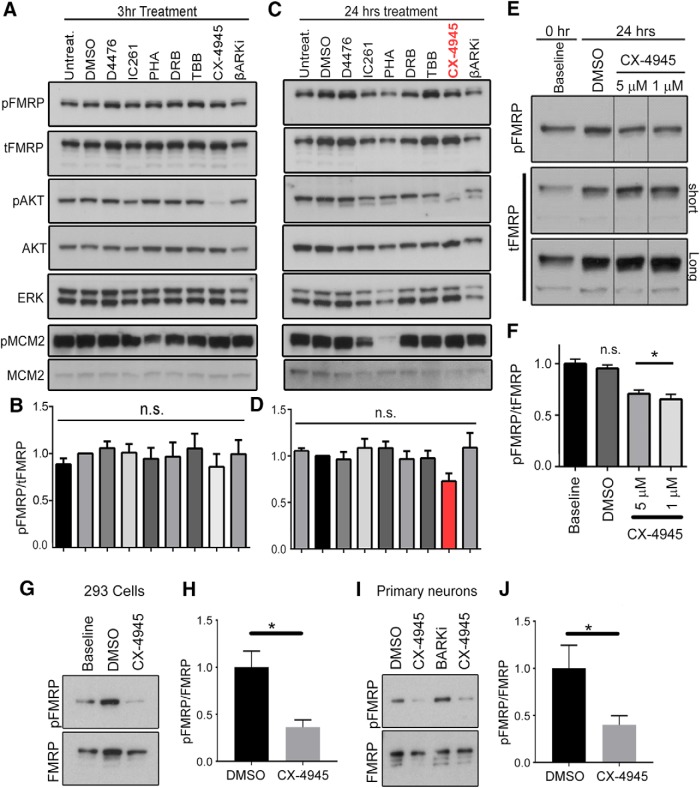
CK2 phosphorylates mammalian FMRP S499. ***A***, Immunoblots for 3-h treatment of N2a cells with vehicle (DMSO), D4476 (25 µm), IC261 (20 µm), PHA-767491 (5 µm), DRB (50 µm), TBB (25 µm), or βARK (200 µm) followed by Western blot for protein indicated on the left. ***B***, Quantification of pFMRP/tFMRP signal in ***A***. ns, not significant (Kruskal–Wallis one-way ANOVA analysis [*H*(8) = 4.56, *p* = 0.8034], *n* = 4). ***C***, Immunoblot for 24-h treatment of N2a cells with the same agents listed in ***A***. ***D***, Quantification of pFMRP/tFMRP signal in ***C*** (Kruskal–Wallis one-way ANOVA [*H*(8) = 7.239, *p* = 0.5111], *n* = 4, error bars = SEM). ***E***, Baseline, untreated N2a cells were collected at time 0, and the remainder of the cells were treated with either DMSO or CX-4945 (5 or 1 µm) for 24 h. tFMRP and pFMRP S499 signals increased in DMSO-treated samples; however, only tFMRP increased in CX-treated samples, thereby causing a significant reduction in relative FMRP S499 phosphorylation. All immunoblot signals are from the same membrane; however, intervening lanes have been removed for clarity. ***F***, Quantification of pFMRP S499 to tFMRP ratio from ***D***; one-way ANOVA, *n* = 4, error bars = SEM, *p < 0.05. ***G***, HEK293 cells were collected at baseline (time 0) or treated for 24 h with DMSO or CX-4945. ***H***, CX-4945 significantly reduced FMRP S499 phosphorylation compared with DMSO (one-tailed Mann–Whitney test, *p* = 0.0143). ***I***, ***J***, Mouse cortical neurons at 7 d *in vitro* treated with 1 µm CX-4945 for 24 h exhibited a significant reduction in FMRP S499 phosphorylation compared with DMSO-treated neurons (one-tailed Mann–Whitney test, *p* = 0.05).

To identify which of the seven kinases was responsible for physiologic phosphorylation of FMRP S499, we performed a pharmacology-based kinase inhibitor screen in N2a cells. We used the following kinase inhibitors with the primary kinase that is inhibited by the pharmacological agent denoted in subscript: D4476_CK1_, IC261_CK1_, PHA-767491_Cdc7/CDK9_, DRB_CDK7_, TBB_CK2_, CX-4945_CK2_, and βARKi_GRK2_. Because many of these inhibitors are capable of inducing cell death after prolonged exposures, N2a cells were maintained in 10% serum (instead of the 5% medium). Surprisingly, none of the inhibitors induced statistically significant dephosphorylation of FMRP after 3 h of treatment (Fig. 2*A*, *B*). We then extended our pharmacological treatment to 24 h. In a multiplexed experiment, none of the inhibitors showed a significant decrease in the proportion of phosphorylation FMRP when analyzed by nonparametric one-way ANOVA (Fig. 2*C*, *D*). However, we noted that the potent CK2 inhibitor CX-4945 showed a visibly larger decrease in the ratio of phosphorylated FMRP S499 over tFMRP ([p/t]FMRP) compared with nearby lanes (Fig. 2*C*, *D*; CX-4945 bar emphasized in red). Although 4,5,6,7-tetrabromobenzotriazole (TBB; 25 µm), another CK2 inhibitor, did not alter [p/t]FMRP, this was likely because CK2 activity was not sufficiently inhibited as noted by residual phosphorylation of the CK2 substrate AKT S129 (AKT S129 is a well-validated readout of CK2 inhibition secondary to CX-4945 treatment). Notably, CX-4945 is ∼40 times more potent than TBB (Cozza et al., 2012) and more than 1000 times more potent than 5,6-dichloro-1-β-D-ribofuranosylbenzimidazole (DRB), which was used in a prior study (Narayanan et al., 2008). We verified that, in general, each kinase inhibitor downregulated phosphorylation of a substrate known to be affected by that kinase (Fig. 2*A*, *C*, representative positive control of decreased pMCM2 S^40/41^/total MCM2 after PHA treatment). In addition to pharmacological inhibition of CK2, we attempted siRNA-mediated knockdown for CK2a1, CK2a2, or both CK2 alpha subunits together; however, all conditions induced massive cell death, thereby confounding interpretation of the ratio of pFMRP/tFMRP (data not shown). This finding was consistent with the fact that CK2 is known to be essential for cell survival (Turowec et al., 2011).

Because CX-4945 efficiently inhibited CK2 activity (as indicated by decreased phospho-AKT S129), elicited an observable decrease in pFMRP S499 in N2a cells, and had been reported to phosphorylate an analogous serine in *Drosophila*, we decided to investigate CK2 further. Given that 24 h of CX-4945 treatment was required to elicit an apparent reduction in FMRP S499 phosphorylation in N2a cells, we suspected that CK2 inhibition impairs phosphorylation of newly translated FMRP rather than reducing phosphorylation of existent FMRP. If true, after 24 h we would expect to see an increase in tFMRP but not pFMRP S499 in CX-4945–treated cells compared with baseline (time 0), leading to a decrease in [p/t]FMRP ratio. To compare the levels of [p/t]FMRP over time, isodense N2a cells were plated simultaneously and lysed in the same volume of buffer at different time points. Because the doubling time of N2a cells in our conditions is 8–12 h, the amount of tFMRP per microliter lysate is also expected to increase over time; the tFMRP level was not quantified because normalization to an also-increasing loading control washes out the real increase in total protein content that occurs over time. We directly tested the hypothesis related to CX-4945’s effect on the phosphorylation of newly translated FMRP by collecting N2a cell lysates at time 0 and a second batch of N2a cells after 24-h treatment with CX-4945 (at both 1 and 5 μm) or DMSO. Consistent with our hypothesis, CX-4945 produced a significant decrease in [p/t]FMRP compared with controls (one-way ANOVA, *n* = 4; Fig. 2*E*, *F*).

To test whether CK2 also phosphorylates FMRP in other mammalian cells, we treated HEK293 cells with CX-4945 and compared 24 h of treatment to baseline and observed a significant decrease in [p/t]FMRP (one-tailed Mann–Whitney test, *p* = 0.0143; Fig. 2*G*, *H*). To extend our N2a and HEK293 cell findings to neurons, we treated cultured murine cortical neurons at 7 d *in vitro* with CX-4945 for 24 h and observed a significant decrease in [p/t]FMRP even without comparison to baseline, presumably because of a more rapid turnover of FMRP in neurons (one-tailed Mann–Whitney test, *p* = 0.05; Fig. 2*I*, *J*).

**Figure 3.**
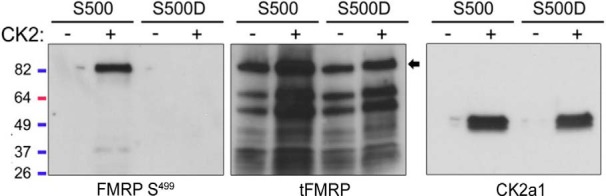
CK2 phosphorylates mammalian FMRP S499 in vitro. rFMRP S500 or S500D was incubated with or without recombinant CK2 for 30 min. Samples were resolved by SDS-PAGE and probed with pFMRP S499, tFMRP, or CK2a1 antibodies. Only rFMRP S499 incubated with CK2 showed a positive phosphosignal.

Given the modest reduction in FMRP S499 signal after 24-h treatment of N2a cells with the CK2 inhibitor CX-4945, we confirmed that CK2 can phosphorylate FMRP at S499/500 in our own hands (as opposed to the Kinexus kinase assay). We performed an in-house kinase assay using recombinant CK2 and rFMRP S500 or S500D. FMRP S500D was included because some phosphoantibodies are known to recognize phosphorylation sites outside of the target epitope. FMRP S500D is not recognized by our phospho-FMRP antibody (Fig. 3), but the aspartic acid preserves the negative charge normally due to phosphorylation at that site. We hypothesized that if CK2 phosphorylated a site outside of FMRP S499/500 that is recognized by our antibody, we would detect a signal after S500D incubation with active CK2. Although the kinase assay with CK2-rFMRP S500 produced a strong signal by SDS page, there was no detectable signal in the FMRP S500D condition or in the absence of ATP (Fig. 3). These data support our previous findings and indicate that the phosphosignal produced by the pFMRP antibody after CK2-mediated phosphorylation is due specifically to phosphorylation of S499/500.

### Washout of the CK2 blocker CX-4945 leads to FMRP rephosphorylation within minutes

**Figure 4.**
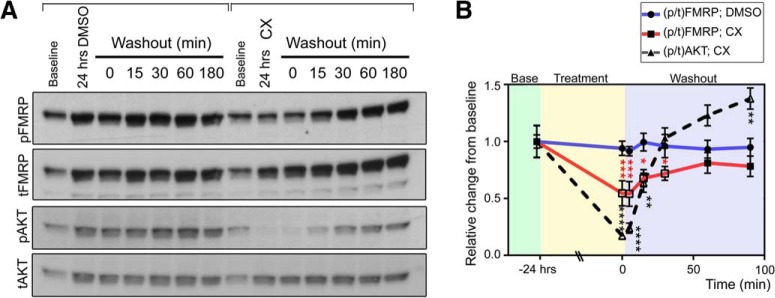
FMRP S499 rephosphorylation kinetics after CX-4945 treatment and washout mirrors a known CK2 target. ***A***, N2a cells were collected either at baseline or 24 h after DMSO or CX-4945 treatment. Additional samples were collected after 24-h treatment and washout of DMSO or CX-4945 for different time periods. ***B***, Quantification of [p/t]FMRP or [p/t]AKT compared with baseline. Changes in ratios were quantified by two-way ANOVA followed by Dunnett’s multiple comparisons post hoc test, *n* = 4 per data point, error bars = SEM. ****p < 0.0001; ***p < 0.001; *p < 0.05.

Because such protracted CK2 inhibition by CX-4945 is required to elicit a reduction in [p/t]FMRP, we wondered whether decreased S499 phosphorylation in the presence of CX-4945 was due to a direct decrease in CK2 activity toward FMRP or indirectly via altered production or degradation of another molecule responsible for direct FMRP phosphorylation. We hypothesized that if the effect of CK2 inhibition were due to direct action on FMRP S499, then washout of CX-4945 would elicit a rephosphorylation of FMRP S499 with kinetics similar to those of a known CK2 substrate (AKT S129). To address this question, we treated N2a cells with either DMSO or CX-4945 (5 µm) for 24 h followed by replacement of the medium to wash out the agent. The first set of N2a cell lysate was collected before drug treatment to establish baseline [p/t]FMRP. A second set of N2a cells was then treated with either DMSO or CX-4945 for 24 h. After 24 h, the second set of N2a cell lysate was collected to determine [p/t]FMRP after 24 h of DMSO or CX-4945 treatment. Finally, after a 24-h treatment with either DMSO or CX-4945, these agents were washed out, and a third set of lysates was collected at 15, 30, 60, or 180 min post-washout. As in prior experiments, we found that between 0 and 24 h tFMRP signal increased in both DMSO- and CX-4945–treated samples, indicating preserved protein translation. In DMSO-treated samples, pFMRP increased in parallel with tFMRP, whereas this increase was significantly blocked by CX-4945 treatment (two-way ANOVA followed by Dunnett’s multiple comparisons post hoc, *n* = 4; Fig. 4*A*). As such, [p/t]FMRP remained stable in DMSO-treated N2a cells, but decreased in CX-4945 cells over 24 h. When we washed out CX-4945, [p/t]FMRP was restored to basal levels in less than 60 min (Fig. 4*B*). This time scale was similar to rephosphorylation of the CK2 substrate AKT S129, which occurred in about 30 min (Fig. 4*A*, *B*).

### CK2 predominantly phosphorylates newly translated FMRP

**Figure 5.**
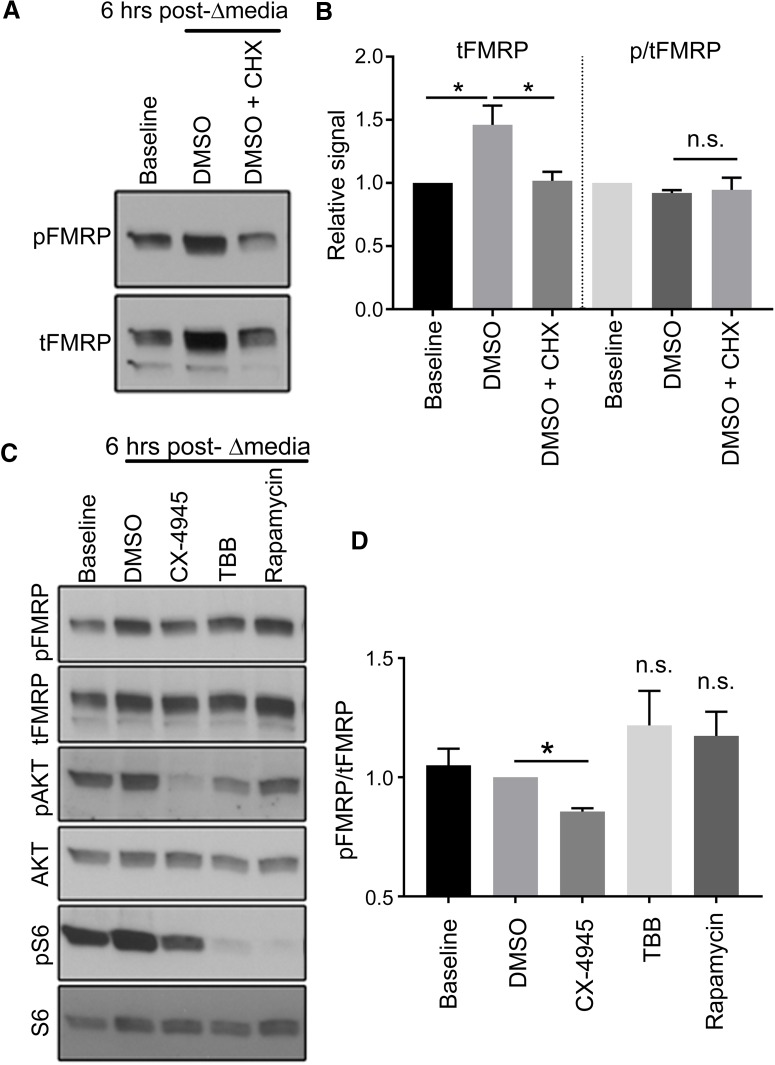
CK2 phosphorylates primarily newly translated FMRP. ***A***, Baseline N2a cells were collected without treatment. The remainder of N2a cells were treated with DMSO or DMSO with cycloheximide for 1 h. After 1 h, the medium was replenished with fresh medium containing DMSO or DMSO with cycloheximide, and cell lysates were collected 6 h later. ***B***, Bar graphs of the relative tFMRP and pFMRP signals at baseline, 6 h after media change + DMSO, and 6 h after media change + DMSO + cycloheximide. Total FMRP was significantly increased 6 h after media change (one-way ANOVA [*H*(4) = 6.736, *p* = 0.0194], *n* = 4), and this increase was blocked by treatment with cycloheximide. The relative pFMRP levels were not significantly different in any of the three conditions (one-way ANOVA [*H*(4) = 4.344, *p* = 0.1131], *n* = 4). ***C***, Baseline cells were collected without treatment, and the remainder of cells were treated with above agents for 1 h. After 1 h, cells were replenished with fresh medium, and cell lysates were collected 6 h later. ***D***, Bar graphs of pFMRP/tFMRP under the different conditions. There was a statistically significant difference of pFMRP/tFMRP between treatment groups and compared with the DMSO control group (one-way ANOVA [*H*(3) = 11.22, *p* = 0.0009]). Dunn’s post hoc multiple comparisons test was not significant between any group and the DMSO control group. However, given the a priori Bayesian hypothesis that CX-4945 decreases pFMRP S499, we performed a nonparametric *t* test between DMSO and CX-4945 groups and found a significant decrease in pFMRP S499 due to 6-h CX-4945 treatment after media change (*p* = 0.0143, *n* = 4).

In the previous experiments, we observed that both pFMRP and tFMRP signals increased over time (time 0 compared with 24-h treatment in DMSO or untreated; Fig. 2) and during washout of CX-4945 when fresh medium is added to the culture well (Fig. 4). As such, we hypothesized that fresh medium induces the production of newly translated FMRP. Indeed, we found that changing N2a cell media 6 h before lysate collection resulted in significantly elevated tFMRP levels compared with baseline (*p* < 0.05, one-way ANOVA, *n* = 4; Fig. 5*A*, *B*). Importantly, this increase in tFMRP was blocked by cycloheximide (30 μg/ml), indicating that the increase in tFMRP was due to an increase in translation rather than decreased protein degradation (Fig. 5*A*, *B*). Consistent with previous experiments, pFMRP S499 increased in parallel with tFMRP such that [p/t]FMRP remained unchanged (Fig. 5*B*).

To test whether the increase in FMRP S499 of newly translated FMRP is CK2 dependent, we treated N2a cells with a series of kinase inhibitors: CX-4945_CK2_ (5 µm), TBB_CK2_ (100 µm), and the negative control rapamycin_mTORC1_ (20 nm). In contrast to previous experiments in which <24-h treatment was insufficient to elicit a significant decrease in [p/t]FMRP (Fig. 2*A*), we found that inhibiting CK2 for 6 h after medium change (using CX-4945) significantly reduced [p/t]FMRP (*p* < 0.05, *t* test, *n* = 4 per condition; see details of the statistics in Fig. 5*C*, *D*). The effect of TBB was insignificant compared with CX-4945, which is consistent with the lesser degree of CK2 inhibition indicated by phospho-AKT S129. Rapamycin did not produce a visible or statistical effect on [p/t]FMRP, as previously reported (Bartley et al., 2014). In that study, DHPG-mediated translation of FMRP produced parallel increases in pFMRP and tFMRP within 2–5 min, indicating that FMRP is peritranslationally phosphorylated.

Taken together, these data indicate that CK2 is a direct physiologic kinase for mammalian FMRP. In addition, these data further suggest that CK2-mediated reduction of FMRP S499 phosphorylation is dependent on the degree of CK2 inhibition and the rate of FMRP production.

### S499 phosphorylation promotes secondary phosphorylation of FMRP

Ceman et al. (2003) demonstrated that phosphorylation of FMRP is preserved when S499 is mutated to the phosphomimetic amino acid aspartic acid (D499; Ceman et al., 2003). Conversely, phosphorylation of FMRP is nearly absent when S499 is mutated to a neutral amino acid, alanine (A499), that cannot be phosphorylated. These data suggest that phosphorylation of S499 promotes secondary phosphorylation of FMRP.

**Figure 6.**
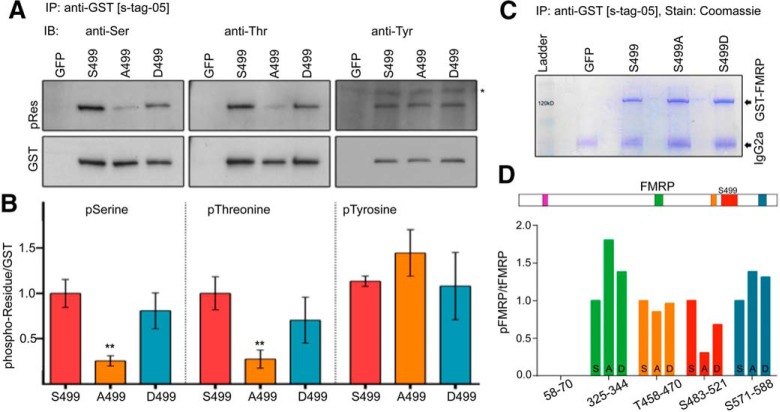
FMRP S499 phosphorylation promotes secondary FMRP phosphorylation on serine/threonine residues. ***A***, Immunoblots (IB) for anti-pSer (left), anti-pThr (middle), and anti-pTyr (right) after immunoprecipitation with anti-GST for the different FMRP constructs (S499, S499A, and S499D). pRes, phosphoresidue. ***B***, Quantification of phosphoresidue over GST blots. **p < 0.01 compared with S499 pRes by one-way ANOVA, *n* = 4 per data point, error bars = SEM. ***C***, Post-IP eluates were separated by SDS-PAGE and Coomassie stained overnight. The GST-FMRP and heavy antibody chain bands are indicated by the arrows on the right. The ∼120-kD band corresponding to the size of GST-FMRP was excised for phosphoproteomic analysis. ***D***, The rectangle above represents FMRP and indicates the location of the identified phosphopeptides after phosphoproteomics. The graph below shows the relative phosphopeptide to total peptide ratio normalized to GST-FMRP S499 and indicates that the proportion of phospho-to-total peptide ratio is selectively decreased for peptide S483–521 in the GST-FMRP A499 condition. The bars for the peptides corresponding to aa 58–70 have been omitted given that their low abundance led to widely spread results that precluded easy representation on the bar graph. The relative abundances for the peptide corresponding to aa 58–70 were GST-FMRP S499 = 1, GST-FMRP A499 = 6.96, and GST-FMRP D499 = 3.03. Note that the overall abundance of the aa 58–70 phosphopeptide relative to all FMRP peptides was <0.04%.

To examine whether S499 promotes additional FMRP phosphorylation, we transfected N2a cells with S499 mutant N-terminal GST-FMRP recombinant constructs. Forty-eight hours after transfection, GST-FMRP S499, A4999, and D499 were isolated by GST-immunoprecipitation under stringent conditions, resolved by SDS-PAGE, and immunoblotted with phosphoserine/phosphothreonine preferential antibodies as well as a phosphotyrosine-specific antibody. We found that mutation of FMRP S499 to alanine produced a significant decrease in the phosphosignal produced by phosphoserine and threonine but not phosphotyrosine antibodies, indicating that S499 phosphorylation primarily modulates the phosphorylation of serine or threonine residues (*p* < 0.01, one-way ANOVA, *n* = 4; Fig. 6*A*, *B*).

Importantly, phosphoserine and phosphothreonine antibodies are known to cross-react with phosphothreonine and phosphoserine, respectively. Because phosphothreonine and phosphoserine signals were reduced to the same degree, and because these antibodies are not singularly specific, further experiments were performed with the phosphothreonine antibody alone, given that it was technically easier to work with. As such, we cannot make assertions as to whether the phosphorylation changes we see with our phosphothreonine antibodies are due to altered serine or threonine FMRP phosphorylation.

### S499-dependent phosphorylation is local to S499

To identify the FMRP S499–dependent phosphoregion, we used mass spectroscopy. GST-FMRP constructs S499, D499, and A499 were overexpressed in N2a cells and isolated by anti-GST immunoprecipitation (IP) under stringent conditions (1 m NaCl). EGFP was used as a negative control. Post-IP eluates were separated by SDS-PAGE and visualized by Coomassie staining. Coomassie revealed that the major protein present in eluents was a ∼115-kD protein corresponding to the expected size of GST-FMRP (absent in the control lane) and antibody chains (Fig. 6*C*). The 115-kD bands were excised from the gel and subjected to trypsin digestion followed by phosphoproteomic analysis.

For quality control, initial spectral searches were performed against the entire Mascot proteome for all species. These scans were performed before phosphopeptide analysis to ensure that quantified phosphopeptides likely originated from FMRP rather than from contaminating proteins. Using exponentially modified protein abundance index scoring, we found that FMRP and the GST peptides are the most abundant peptides in our samples (Ishihama et al., 2005). When contaminants common to all samples (such as keratin and albumin) are ignored, FMRP proteins are 31–84 times more abundant than coeluting proteins (data not shown). These data indicate that the phosphosignal detected by our phosphoantibodies is primarily due to the phosphorylation of FMRP.

Given that FMRP peptides represented the bulk of peptides in our samples, we restricted our phosphoproteomic search of the spectra from these same samples to a custom database limited to GST-FMRP S499, GST-FMRP D499, and GST-FMRP A499. For each sample, phosphoregions were identified and, using spectral counts, the ratio of phosphorylated to total peptides for that region was determined (Lundgren et al., 2010). For each sample, the ratio was normalized to the corresponding ratio for the same phosphopeptide calculated for GST-FMP S499. Using this approach, of the five phosphopeptides identified only one (aa 483–521) exhibited a decreased ratio of phosphorylated to total peptides (∼75.9% reduction for A499 and ∼14.6% reduction for D499; raw data and Excel analyses are accessible at https://figshare.com/s/5a5d2016eaa41692fc09), consistent with our immunoaffinity data and the radiolabeling data from Ceman et al. (2003) (Fig. 6*D*). Taken together, these data suggest that FMRP S499 phosphorylation promotes secondary phosphorylation of nearby regions of FMRP.

### S499 phosphorylation may be permissive for secondary modulation of serine/threonine phosphorylation by PP2A and mGluR-I

It was previously reported that neither the mGluR-I agonist DHPG nor the PP2A inhibitor okadaic acid alters the ratio of phosphorylated FMRP S499 to total FMRP (Bartley et al., 2014). Given prior studies indicating that FMRP phosphorylation is influenced by mGluR-I signaling and PP2A activity using radiographic methods (Narayanan et al., 2007; Narayanan et al., 2008), we hypothesized that these pathways regulate secondary phosphorylation of FMRP rather than S499 itself.

**Figure 7.**
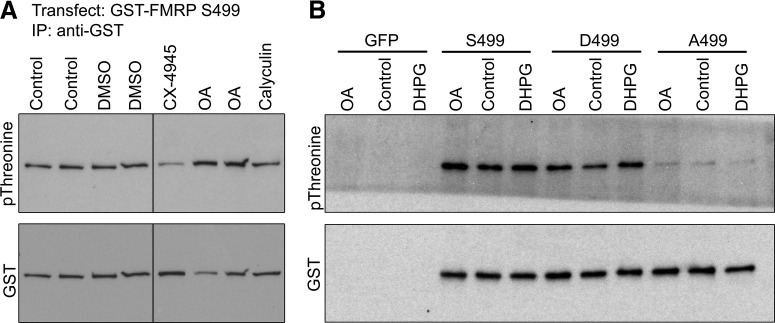
FMRP S499 influences the phosphorylation of other FMRP residues, possibly downstream of PP2A and mGluR-I. ***A***, N2a cells were transfected with GST-FMRP S499 and treated with DMSO, CX-4945, okadaic acid, or calyculin for 6 h. After treatment, FMRP was isolated via GST immunoprecipitation, and samples were probed for phosphothreonine. The same membranes were then stripped and probed for total GST. CX-4945 dramatically decreased the phosphothreonine signal. The PP2A inhibitor okadaic acid dramatically increased the phosphothreonine signal, whereas the PP1 inhibitor had no detectable effect. ***B***, N2a cells were transfected with GST-FMRP S499, D499, and A499 constructs. Cells were treated with okadaic acid for 1 h or DHPG for 2 min before lysis. FMRP was isolated by GST immunoprecipitation and probed for phosphothreonine. Okadaic acid and DHPG increased phosphothreonine signals for S499 and D499 compared with DMSO control, whereas A499 phosphorylation was unresponsive to either treatment.

We first tested whether PP2A modulates FMRP phosphorylation by treating N2a cells transfected with GST-FMRP S499 with okadaic acid for 6 h after medium change and probing IP isolates with our phosphothreonine antibody. Additionally, we treated GST-FMRP S499–transfected cells with calyculin to test for alternative phosphatase activity (PP1) toward FMRP. Finally, we also examined the effect of CX-4945 treatment as a positive control. As expected, CX-4945 produced a dramatic reduction of phosphorylation, presumably owing to both reduced S499 phosphorylation and secondary phosphorylation. Okadaic acid, but not calyculin, resulted in a robust increase in the FMRP phosphothreonine signal, suggesting that PP2A is a major FMRP phosphatase (*n* =2; Fig. 7*A*).

Next, to more directly test whether S499 phosphorylation regulates secondary FMRP phosphorylation downstream of PP2A or mGluR I pathways, we treated GST-A499– and GST-D499– transfected N2a cells with okadaic acid (1 h instead of 6), DHPG (2 min), or vehicle (DMSO or water, respectively) and probed isolates with an anti-phosphothreonine antibody. We found that both okadaic acid and DHPG treatment increased the phosphorylation signal for FMRP D499 but not for FMRP A499 (Fig. 7*B*).

**Figure 8.**
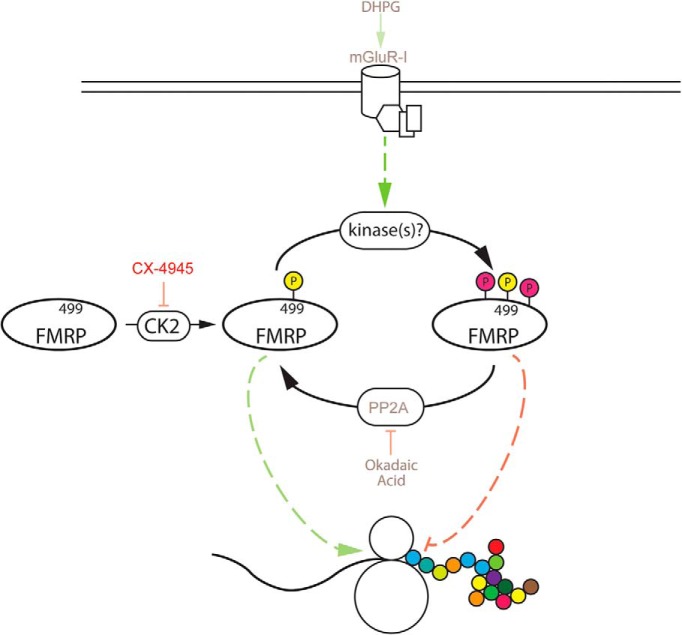
Alternative model for FMRP phosphorylation and regulation of translation. FMRP is first phosphorylated by CK2 on S499. FMRP S499 phosphorylation is permissive for secondary phosphorylation of FMRP on serine/threonine residues, presumably downstream of mGluR-I, by unknown kinases. Secondary phosphorylation of FMRP is counteracted by PP2A-mediated dephosphorylation. The dotted green and red lines are provisional and indicate that the relationship between FMRP’s phosphorylation status and protein translation is unknown.

Taken together, these data support an alternative phosphodynamic model of FMRP, in which CK2 phosphorylation of mammalian FMRP S499 precedes and promotes secondary phosphorylation of FMRP via an unknown kinase or kinases; secondary phosphorylation sites are possibly downstream of PP2A/mGluR-I activity (Fig. 8).

## Discussion

Although phosphorylation of serine 499 plays a requisite role in FMRP biology, remarkably little is known about which kinase is responsible for its phosphorylation. It was recently reported that the mechanistic target of rapamycin complex 1 (mTORC1) pathway does not regulate S499 and that previously implicated pathways mGluR-I and PP2A apparently have no effect on the state of S499 phosphorylation (Bartley et al., 2014). Finally, our observation that FMRP S499 and FMRP D499 constructs are functionally equivalent in most studies called into question whether S499 phosphorylation dynamically regulates FMRP function. As such, we first endeavored to identify the kinase responsible for FMRP S499 phosphorylation and investigate whether other residues are regulated by pathways implicated in FMRP function, mGluR-I and PP2A.

To identify the kinase for FMRP S499, we performed an *in vitro* kinase screen of a pool of rationally selected kinases. In total, six of 18 tested kinases showed *in vitro*, site-specific activity toward FMRP. It was previously shown that S6K1 also phosphorylates FMRP S499 *in vitro* despite showing no physiologic activity toward FMRP (Bartley et al., 2014). As such, we screened these six kinases for *in vivo* kinase activity toward FMRP S499 using a panel of overlapping kinase inhibitors. Surprisingly, 3-h-long treatment of N2a cells with a broad array of inhibitors failed to yield a significant decrease in FMRP S499 phosphorylation. Extended 24-h-long treatment of N2a cells demonstrated that the highly potent and specific CK2 inhibitor CX-4945 significantly decreased FMRP S499 phosphorylation. Although our data show that CK2 phosphorylates mammalian FMRP S499, we directly tested only ∼5% of known kinases for activity toward FMRP S499. Nonetheless, many additional kinases were indirectly tested, as staurosporine inhibits nearly 50% of known kinases, and many of the kinase inhibitors used in this study inhibit off-target kinases. Notably, PHA-767491 inhibits the most evolutionarily similar kinase to CK2, Cdc7, but did not reduce FMRP S499 phosphorylation. Taken together, our data identify CK2 as a direct FMRP S499 kinase but do not rule out the existence of other S499 kinases.

Although CK2 had previously been identified as the kinase for a homologous serine in *Drosophila* FMRP, that CK2 is also the FMRP S499 kinase in mammals was not entirely expected for at least two reasons. First, the divergence between *Drosophila* (S406) and mouse (S499) FMRP sequences suggested that although the regions are functionally related, they may be phosphorylated by distinct kinases. Second, CK2 is considered to be a constitutively active kinase that is not thought to be regulated by neuronal activity. Therefore, it was unclear how CK2 might phosphorylate FMRP in response to activity. Moreover, a previous study had reported that CK2 does not phosphorylate murine FMRP S499, based on a persistent phosphosignal after treatment of cultured neurons with the CK2 inhibitor DRB (Narayanan et al., 2008). Despite sequence divergence, constitutive CK2 activity, and reports that CK2 is not the kinase in mouse, we found that mammalian FMRP S499 is phosphorylated by CK2 by using a highly selective and potent kinase inhibitor. We too found that DRB had no effect on pFMRP S499; however, DRB is a very weak CK2 inhibitor and was incapable of dephosphorylating a known CK2 substrate (AKT S129). Additionally, in the previous study, DRB treatment was <6 h, which may be insufficient time to elicit a decrease in the phosphorylation of FMRP S499 in neurons under basal conditions.

Although CK2 is considered to be a constitutively active kinase, we were surprised to find that S499 phosphorylation was quite stable. Under basal conditions, 24 h of CX-4945 treatment was required to elicit a significant decrease in the proportion of pFMRP. That the effect of CX-4945 is observed sooner under conditions of increased FMRP translation suggests that CK2 phosphorylates newly translated FMRP, and once phosphorylated, it remains phosphorylated. Such one-off and persistent phosphorylation has been observed in other proteins, notably DYRK1A (Lochhead et al., 2005) and AKT T450 (Oh et al., 2010), and is thought to be due to a change in conformation that buries the phosphoresidue and obscures it from potential phosphatases. Whatever the cause of persistently phosphorylated FMRP S499, this observation coupled with data from the literature that S499 and D499 are functionally similar led us to hypothesize that phosphorylated S499 is permissive for the secondary phosphorylation of activity-dependent residues.

Using label-free quantification of spectral counts, our mass spectrometry data identify the S499-dependent phosphoregion as a peptide containing S499 itself, which is consistent with work by Ceman et al (2003). We were unable to unambiguously assign phosphoresidues, and our method of protein cleavage (CID) is known to promote phosphate migration from one amino acid to another, further contributing to site ambiguity. Notably, Ceman et al (2003) also used CID, and they too comment on their inability to unambiguously identify additional FMRP phosphorylation sites. Nonetheless, the multiply phosphorylated FMRP peptide that was identified by Ceman et al (2003) at baseline overlaps with the S499-dependent multiply phosphorylated peptide that we identified. Therefore, our data represent the first independent replication and extension of this important finding. We also identify phosphopeptides that are not S499-dependent and three out of four of the peptides (all but the least abundant, aa 58–70) have been observed by others in independent mass spectrometry experiments, as indicated by phosphosite.org.

To evaluate whether phosphorylated S499 influences secondary phosphorylation of additional resides, we pharmacologically inhibited PP2A and stimulated mGluR-I pathways, because these two pathways have been shown to regulate FMRP phosphorylation and function by way of site-nonspecific autoradiography (Narayanan et al., 2007, 2008). We first confirmed that neither PP2A inhibition nor mGluR-I activation alters the phosphorylation of FMRP S499. We then found that both PP2A inhibition and mGluR-I stimulation increase the phosphorylation of GST-FMRP D499 but not GST-FMRP A499. These data warrant independent replication and suggest that FMRP S499 serves as a priming site that is required for the secondary phosphorylation of additional residues regulated by the PP2A and mGluR-I pathways.

In this study, we identified a major mammalian S499 kinase and found evidence that S499 phosphorylation is permissive for and promotes secondary phosphorylation of FMRP. Although we confirmed secondary phosphorylation of FMRP with a phosphothreonine-specific antibody, this antibody exhibits cross-reactivity with ∼20% of phosphoserine residues (unpublished observations), and we therefore cannot make a claim as to whether PP2A and mGluR-I pathways regulate serine or threonine residues on FMRP. A fruitful approach to identifying the sites regulated by these pathways would include phosphoproteomics in negative ion mode, site-directed mutagenesis, and site-specific phosphoantibody generation for immunoaffinity assays.

Taken together, our data support an alternative model of FMRP phosphorylation that is consistent with the published literature. This model posits that FMRP is peritranslationally phosphorylated by CK2, which allows for secondary phosphorylation of secondary residues in an activity-dependent manner. Given that FMRP’s role in activity-dependent protein translation is well described and that phosphorylation of S499 is required for FMRP-dependent inhibition of translation, identification of these secondary phosphosites is likely to yield insight into the mechanism of FMRP-mediated translational inhibition.

## Synthesis

Reviewing Editor: Alfonso Araque, University of Minnesota The decision was a result of the Reviewing Editor Alfonso Araque and the peer reviewers coming together and discussing their recommendations until a consensus was reached. A fact-based synthesis statement explaining their decision and outlining what is needed to prepare a revision is listed below. The following reviewer(s) agreed to reveal their identity: Joaquin Lugo

The reviewers have found merit and interest on the findings reported. However, they also expressed several serious concerns regarding several methodological aspects that need to be addressed. Besides the specific further experimental evidence required (see below their specific comments), the insufficient appropriate statistical analysis and the excessive data not shown need to be addressed. Also, as noted below by one of the reviewers, more convincing evidence for mGluR-I and PP2A regulation of secondary phosphorylation sites on FMRP should be provided.

Reviewer 1

The authors provide an interesting and novel data for the role of CK2 on phosphorylated FMRP and how this process promotes additional phosphorylation of FMRP. The data do appear to provide a bridge between the perceived inconsistencies in the literature. The series of experiments were logical and each experiment nicely provided supporting evidence for their hypothesis. The paper was well-written and was easy to follow.

However, the lack of statistical analyses is a concern but can be easily completed. I would recommend that the analyses be placed in the results and not in the figure legends. In addition in several of the figures the statistical analyses should be included. For example in figure one the two-way ANOVA or independent t-test should be included for figure 1B. This will help to determine the effect size in their manipulation.

In figure 4 and in figure 6 the quantification of the western blots would significantly enhance the conclusions of their study. In figure 6 the authors mentioned a robust increase by OA but did not provide statistical evidence. Since they have quantified the other western blots it was not clear why they did not quantify these. The quantification would enhance the paper.

Another issue is the overuse of (data not shown). Since the authors are proposing novel regulation of pFMRP the negative results, evidence of cell death, and other data would be greatly appreciated. Not all data need to be included but I counted 5 instances. The data could be included in supplemental methods.

Even though I did find some issues in the manuscript I believe the data are interesting and will advance the field.

Reviewer 2.

The authors have shown that a constitutively active kinase, casein kinase (CK) 2 mediates phosphorylation of murine FMRP S499 in in vitro kinase assays, cell lines and primary neurons. Additionally, the authors show data to suggest that primary S499 phosphorylation of FMRP permits phosphorylation of additional residues in response to mGluR-I and PP2A pathways. Because S499 FMRP phosphorylation has been shown to be important for FMRP function in neurons, determining the direct kinase in mammalian cells is important. Previous work from Ceman et al., indicated that there S499 triggers additional phosphorylation of other neighboring residues. Work from Narayanan et al., indicated that mGluR and PP2A regulated phosphorylation of FMRP, as observed with metabolic labelling, and suggested this was S499, but did not conclusively show this. This current work revises and adds detail to the hierarchical phosphorylation of FMRP that is allowed by S499 phosphorylation by CK2. The data indicating the phosphorylation of S499 by CK2 is of good quality and convincing. However, data that mGluR-I and PP2A regulate secondary phosphorylation sites on FMRP is sparse and not really convincing.

Specific concerns:

1. In Figures 5,6 the authors use pThr and pSer blots after IP of FMRP to indicate that mGluRs and PP2A regulate other phosphorylation sites on FMRP that depend on S499 phosphorylation. An issue is the specificity pSer, pThr, pTyr blots signal of immunoprecipitation of GST-FMRP under stringent conditions. Other interacting proteins could be pulled down with the GST-FMRP. A more rigorous and convincing demonstration that PP2a and mGluR regulate phosphorylation of other sites is to use mass spec of GST-FMRP to identify these sites. Mutations and/or phosphospecific antibodies at these secondary phosphorylation sites of FMRP can then be generated to determine the regulation by neuronal activity. Candidate sites may be taken from Ceman et al., who identified FMRP residues hierarchically phosphorylated with S499.

2. Additional evidence for regulation of secondary phosphorylation by mGluR would be to demonstrate the kinase for these secondary phosphorylation sites. As the authors point out, S6K1 does not phosphorylate S499 FMRP as detected by phosphosite specific antibody but (Narayanan et al., 2008) demonstrated that S6K1 phosphorylated FMRP as assessed with metabolic labeling and this relies on S499 phosphorylation. So S6K1 is a good candidate. For such reasons, it is unclear why rapamycin is a negative control for Fig. 4B. This should be better explained.

3. For some results no group data is shown. Therefore, it is difficult to know the reproducibility and reliability of the results or how many times this was replicated. For example, Fig. 3E,F, Fig. 4 and 6. For example, it is difficult to see the changes of pFMRP/tFMRP in CX-4945 treatment.

4. A loading control is required to compare tFMRP protein level between baseline and 24hr treatment.

Minor points:

1. page 8, "ERK1 and p38a activity towards rFMRP could not be determined by SDS-PAGE due to artifact" what is mean by"artifact"?

2. Figure 4B, there is no label on the 6th lane. What is the lane?

3. The bars on Fig. 5B are mislabeled according to the corresponding western blots above. D499 should be A499 and vice versa.

4. There is no pSer, pThr, pTyr antibodies information in Table 1.

5. There are several typos in the manuscript. Proofreading is required.
